# Impact of Coronavirus-2019 on General Health Anxiety Among Students of Iranian Medical Sciences: COVID-19 and Students’ Anxiety

**DOI:** 10.31661/gmj.v11i.2537

**Published:** 2022-12-31

**Authors:** Farzaneh Modaresi, Amir Ansari, Sadaf Abyar, Aliasghar Karimi, Mahboubeh Eslamzadeh, Fatemeh Shekoohi, Hamid Reza Sabet

**Affiliations:** ^1^ Non-Communicable Diseases Research Center, Fasa University of Medical Sciences, Fasa, Iran; ^2^ Student research committee, Fasa University of Medical Sciences, Fasa, Iran; ^3^ Research Center for Neuromodulation and Pain, NAB Pain Clinic, Shiraz University of Medical Sciences, Shiraz, Iran; ^4^ Psychiatry and Behavioral Sciences Research Center, Mashhad University of Medical Sciences, Mashhad, Iran; ^5^ Clinical Research Development Unit, Valiasr Hospital, Fasa University of Medical Sciences, Fasa, Iran; ^6^ Medical Journalism Department, Faculty of Paramedical Sciences, Shiraz University of Medical Sciences, Shiraz

**Keywords:** COVID-19, General Health Anxiety Index, Healthcare Students, Iran, pandemic

## Abstract

**Background:**

During the Coronavirus 2019 (COVID-19) outbreak, Iranian medical sciences students were at higher risk of contracting this virus because they were in infected environments. So, they are predisposed to high levels of anxiety that could worsen their lives. The determent of factors and levels of anxiety could be helpful to reduce anxiety and control its worse effects. Hence, this study aimed to measure the anxiety index and its factors among medical sciences students during the COVID-19 pandemic in Iran.

**Materials and Methods:**

In this cross-sectional study, an online survey was sent to students from 27 medical sciences universities in Iran from 20th December 2020 to 10th March 2021. The online survey consists of the Health Anxiety Inventory (HAI) for measured general health anxiety as well as the baseline characteristics of students.

**Results:**

723 students responded, including 483 (66.8%) females and mean HAI score was 16.76±8.35. Based on our findings, gender, past medical, and drug history were significantly related to the high level of anxiety. However, there was no coloration between HAI scores with age, the field of study, study duration, university location, and attendance in the hospital and/or COVID-19 ward (P˃0.05).

**Conclusions:**

Students with notable past medical history and/or drug history and female students more than others were predisposed to anxiety in a pandemic such as COVID-19. Hence, in a pandemic situation, psychological care should concern them.

## Introduction

The expansion of Coronavirus 2019 (COVID-19) due to its speed of spread made a global health emergency in less than a few months. It also causes several psychological illnesses, including anxiety, fear, depression, avoidance behaviors, irritability, sleep disorder, and post-traumatic stress disorder [1].

According to past studies, epidemics of infectious diseases have profound psychosocial effects on individuals and society [2]. At the individual level,examples are people who experience fear of illness and death, stigma, and helplessness [2]. A short-term review study that investigated 14 studies on healthcare workers' anxiety levels during the COVID-19 pandemic showed high levels of anxiety (2.2%) and depression (14.5%) in healthcare workers that suffered from severe symptoms [3]. Also, Bohlken *et al*. indicated that the age, gender, specialty, and type of work of individuals during the COVID-19 pandemic influenced the severity of the psychological symptoms [3]. Psychological factors play an essential role in the success of strategies such as indiviuals interaction with each other, vaccinations, hand hygiene, and antiviral therapies adopted by general health systems during epidemics and pandemics [4]. Indeed, health anxiety affects these strategies' success or failure [4].

Health anxiety is a multidimensional structure that includes mood, behavioral, perceptual, and cognitive [5]. Epidemiological studies have shown that health anxiety is a common clinical complaint associated with a negative interpretation and a sense of fear in interpreting a person's physical symptoms as to whether it is a common symptom of an unusual and dangerous finding [6][7].

When given information about a disease, individuals with health anxiety show impaired responses and appear to employ inappropriate defensive mechanisms. [8][9]. In other words, persons with health anxiety cause to impose economic costs on the health system for some reasons, e.g., unnecessary medical advice and unreasonable diagnostic tests [10]. Thus, health anxiety has long-term effects on psychological performance and treatment costs. Epidemics of viral diseases and the seasonal outbreak of diseases such as flu significantly affect the attendance of people in hospital emergency wards to visit a doctor [11]. In major epidemics, even when people have only mild symptoms of the disease, they go to inpatient and outpatient medical centers, which could lead to excess and unnecessary pressure on the healthcare system [11].

The pandemic of COVID-19 has led to a lot of psychological pressure on the medical staff. Due to their presence in high-risk environments, these individuals are faced with concerns about their infection and their relatives and families [12]. They may reduce and/or discontented communication with others for a long time-which considering their heavy duties these people- could lead to insomnia, anger, and anxiety [12]. In this situation, medical staff, including medical students and related fields, are predisposed to experience higher anxiety disorders due to involvement in more high-risk environments as well as more information about diseases [13].

The level and factors of anxiety among medical science students in Iran have not yet been sufficiently examined during the COVID-19 pandemic. Hence, the current study aims to measure the anxiety index and its factors among Iranian medical sciences students during the COVID-19 pandemic.

## Materials and Methods

This cross-sectional study was conducted on students from medical universities across the 27 universities in Iran from 20^th^ December 2020 to 10^th^ March 2021.

### Ethical Considerations

This study was approved by the Ethics Committee of the Research Vice-Chancellor of Fasa University of Medical Sciences (approval code: IR.FUMS.REC.1399.039). All respondents have informed consent, and authors were assured that all data are confidential and published anonymously.

### Sample Size Calculation

The sample size was initially calculated at 664 with 99% power and 5% error based on following the formula:


n = \frac{z_{1}^2 -\alpha/2 \times P(1-P)}{d^2}

Hence, by considering a 15% drop in sample volume, the final sample size was 996 students.

### Data Collections

Data collection was conducted through an electronic survey via the online Persian version (powered by Google^®^ form) of the Health Anxiety Inventory (HAI) that by simple random sampling methods, was sent to 1000 students from 27 medical universities in Iran. The HAI is a validated screening instrument based on the core symptoms of anxiety that Salkovskis and Warwick designed in 2002 to measure individuals' health anxiety (Cronbach's alpha coefficient and its validity were reported as 0.7 to 0.8 and 0.63, respectively) [14]. Also, Nargesi *et al*. [15] validated the Persian version of HAI (Cronbach's alpha for the whole inventory was 0.75 mentioned that scores less than26=low health anxiety, between 26 and 35=mid-health anxiety, and scores equal to or more than 36=high health anxiety [15]

HAI includes 18 items, each with four domains, and each domain consists of the description of the person's health and illness components as a declarative sentence that the respondent should choose one of the options that best describes them (each respond scored from 0 to 3) [15]. Accordingly, the highest and lowest score is 54 and zero, respectively [15]. The health anxiety of individual was considered as low (score lower than 26), mild (score 26 to 35), and high (score 36 and more) [15].

Moreover, the subjects responded to questions regarding basic characteristics such as age, gender, past medical history (including cardiovascular, neurological, autoimmune, immune deficiency, diabetes mellitus, malignancy, and psychological disorders). Also, the history of medication intake, the field of study, duration and location of work were collected.

### Statistical Analysis

Data were presented as mean±standard deviation and analyzed by T-test, F-test, and Pearson correlation test using IBM SPSS Statistics for Windows, version 22 (IBM Corp., Armonk, N.Y., USA). The significance level was considered as P=0.05.

## Results

**Table:** Table-1. The Correlation between HAI Score and Baseline Characteristics of Participants

**Variables**	**n (%)**	**HAI Score (Mean**± **SD)**	**Anxiety level**	**P-value**
**Gender**				
Male	239	15.55±8.51	Low	0.006
Female	484	17.37±8.21	Mild	
**Past medical history**				
Yes	79	18.77±8.5	Mild	0.02
No	644	16.51±8.3	Mild	
**Drug history**				
Yes	79	19.53±8.63	Mild	0.002
No	644	16.42±8.25	Mild	
**Working in hospital**				
Yes	204	17.44±8.37	Mild	0.16
No	519	16.49±8.33	Mild	
**Working in COVID-19 wards **				
Yes	68	15.75±8.61	Low	0.29
No	655	16.86±8.32	Mild	
**Field of study**				
Medicine	378	16.95±8.18	Mild	
Nursing	74	16.28±9.08	Mild	
Medical laboratory sciences	58	14.94±6.01	Low	
Special residency	50	15.78±6.78	Low	
Dentistry	41	18.87±8.66	Mild	
Anesthesia assistant	30	16.9±9.08	Mild	0.17
Surgical technology	30	15.3±7.58	Low	
Public health	27	16.29±11.66	Mild	
Pharmacology	22	18.95±8.18	Mild	
Radiology assistant	8	15.87±13.53	Low	
Midwifery	5	25±6.04	Mild	

**HAI:** Health anxiety inventory;

**Table:** Table-2. The Correlation between HAI Score and Baseline Characteristics of Participants

**University**	**Frequency**		**HAI Score**		**P-value**
		**(Mean** **±** **SD)**	**Minimum**	**Maximum**	
Fasa UMS	221	15.5±6.81	0	51	
Bushehr UMS	111	18.1±10.75	0	50	
Shiraz UMS	103	16.7±8.36	3	52	
Zanjan UMS	69	18.9±9.95	0	53	
Yazd UMS	62	16.38±7.68	1	44	
Mashhad UMS	29	16.2±8.33	4	44	
Hormozgan UMS	22	18.1±8.16	8	37	
Yasoj UMS	20	15.4±6.36	5	31	
Bojnord UMS	18	19.66±8.08	6	34	
Jahrom UMS	15	18.1±5.73	8	27	
Iran UMS	10	17.5±6.11	7	26	
Ghazvin UMS	8	14±6.34	2	23	
Mazandaran UMS	6	14.8±14.83	7	22	
Kerman UMS	5	11.8±3.42	6	15	
Zahedan UMS	4	14.7±4.57	9	20	0.104
Birjand UMS	3	18.8±7.3	13	27	
Tehran UMS	2	17.5±10.6	10	25	
Shahrood UMS	2	13	13	13	
Rafsanjan UMS	2	9±8.48	3	15	
Arak UMS	2	26.5±27.57	7	46	
Ahvaz UMS	2	21±7.07	16	26	
Shahid Beheshti UMS	1	34	34	34	
Lorestan UMS	1	23	23	23	
Kermanshah UMS	1	21	21	21	
Hamedan UMS	1	11	11	11	
Bam UMS	1	11	11	11	
Alborz UMS	1	15	15	15	
Missing	1	-	-	-	
**Total**	723	16.76±8.35	0	53	

**HAI:** Health anxiety inventory; **UMS:** University of medical sciences

Overall, 723 students filled out the HAI form. The mean age of participants was 23.05±4.05 years and 483 (66.8%) were females. The total mean HAI score was 16.76±8.35, and 644 (89.1%), 53 (7.3%), and 26 (3.6%) students had low, mild, and high levels of anxiety, respectively. As shown in Table-1, the mean HAI score was significantly higher in females (P=0.006). The differences between HAI score and past medical and drug history were significant (Table-1, P=0.02 and P=0.002, respectively).

Although 272 students were required to work in the hospital and COVID-19 ward; however, their HAI score was not significantly different from other students (Table-1)

The mean duration of the study was 3.17±1.94 years, and the lowest and highest HAI scores were observed in students of medical laboratory sciences and midwifery; however, there was no any correlation between HAI score and field of study (Table-1, P=0.17).

According to Table-2, the minimum and maximum HAI scores were observed among the students of Rafsanjan (9) and Shahid Beheshti (34) universities, respectively. However, our findings indicated that there was no significant correlation between HAI scores and the university of students (Table-2).

## Discussion

During the COVID-19 pandemic, Iranian medical science students were predisposed to be infected with this virus. So, many feared they would be infected in the hospital and transmit it to their families and relatives.

Recently, Bahmaei *et al*. [16] revealed that HAI score among the students was 34.8 to 41.7, while this score in our study was 16.76, indicating that in contrast to our finding, most of the sutudets have high levels of anxiety [16]. This difference may be related to vaccine news and/or increased information about COVID-19.

According to our findings, females were more concerned and anxious about their health than male students, which is consistent with the findings of Al-Rabiaah *et al*. in Saudi Arabia [17]; however, Bahmaei *et al*. [16] found no significant difference in terms of gender.

Also, positive past medical history increases students' concerns and anxiety. Moreover, the drug history increases students anxiety levels, so these findings are also consistent with the results of Al-Rabiaah *et al*. [17], Akbas *et al*. [18], Safa *et al*. [19], and Shahdad *et al*. [20].

However, our results demonstrated that age, the field of study, duration of the study, university location, and section of work (e.g., COVID-19 ward) showed no significant effect on the level of anxiety. These findings are similar to the results of Bahmaei *et al*. [16]

According to the results, the lowest and the highest average of total HAI belongs to the students of Rafsanjan and Shahid Beheshti universities of medical sciences, which showed a wide range of changes in HAI scores. However, the average overall HAI of all students of Shahid Beheshti University was calculated as 16.76, which indicated that the health anxiety level was relatively low during the COVID-19 pandemic.

## Conclusion

According to the current study, it is suggested that in the pandemic situation, female students should be given special attention with purposeful counseling and improvement of mental and psychological conditions. Also, students with a positive past medical history and who have intake drugs for these illnesses should be encouraged to reduce the stress and anxiety associated with the disease. Also, preventive measures and appropriate training should be provided for safe confrontation with infectious and contagious diseases to reassure students who are required to attend medical centers. It is recommended that the universities exchange and share their experiences continuously in dealing with dangerous conditions.

## Conflict of Interest

The authors declared no conflict of interest. Also, Aliashghar Karimi, one of the article's authors, is the "editor in chief" of the GMJ journal. Based on the journal policy, this author was excluded entirely from any review process of this article.
